# Desmoplastic small round cell tumor of the parotid gland-report of a rare case and a review of the literature

**DOI:** 10.1186/s13000-019-0825-1

**Published:** 2019-05-18

**Authors:** Kanako C. Hatanaka, Emi Takakuwa, Yutaka Hatanaka, Akira Suzuki, Satoshi IIzuka, Nayuta Tsushima, Tomoko Mitsuhashi, Shintaro Sugita, Akihiro Homma, Shojiroh Morinaga, Tadashi Hashegawa, Yoshihiro Matsuno

**Affiliations:** 1 Department of Surgical Pathology, Hokkaido University Hospital, N14W4, Kita-ku, Sapporo, Japan; 2 Department of Pathology, KKR, Sapporo Medical Center, 1-6, hiragishi, Toyohira-ku, Sapporo, Japan; 3 Department of otorhinolaryngology, Hakodate Central General Hospital, 33-2, Honcho, Hakodate, Japan; 4 Department of Surgical Pathology, Sapporo Medical University School of Medicine, S1W16, chou-ku, Sapporo, Japan; 5 Department of Otolaryngology-Head & Neck Surgery, Faculty of Medicine and Graduate School of Medicine, Hokkaido University, N15W7, Kita-ku, Sapporo, Japan; 6 Department of Diagnostic Pathology, Hino Municipal Hospital, 4-3-1, Tamadaira, Hino, Tokyo, Japan

**Keywords:** Desmoplastic small round cell tumor, Salivary gland, WT1, C-terminal region, 3′/5′ expression imbalance assay

## Abstract

**Background:**

Desmoplastic small round cell tumor (DSRCT) is a rare soft tissue tumor that generally involves the retroperitoneum, pelvis, omentum and mesentery in younger patients. However, extra-abdominal DSRCT is very rare.

**Case presentation:**

A 49-year-old Japanese man noticed a mass in the right parotid gland. Ultrasound examination revealed a solid tumor about 2 cm in diameter. Computed tomography (CT) of the whole body revealed no other tumors or lymph node swelling. Superficial parotidectomy was performed. Histologically, the tumor was composed of various-sized tumor cell nests in an abundant fibromyxoid and collagenous background. The tumor cells were small to medium-sized. Immunohistochemistry showed that the tumor cells were immunoreactive for epithelial markers and desmin. They also showed strong nuclear staining with a Wilms tumor 1 (WT1) antibody detecting the C-terminal region (C-WT1), but not the N-terminal region (N-WT1). We also performed 3′/5′ expression imbalance assay based on reverse transcription polymerase chain reaction (RT-PCR) to determine whether aberrant WT1 gene expression was present. This tumor was found to lack 5′-regional expression of the WT1 gene, as well as immunoreactivity with the N-WT1 antibody. Finally, fluorescence in situ hybridization (FISH) and RT-PCR analyses revealed the presence of a gene showing fusion between exon 7 of EWSR1 and exon 8 of WT1. The tumor was diagnosed as a DSRCT of the right parotid gland. The patient has been followed for 3 years without recurrence or metastasis.

**Conclusions:**

Although DSRCT in the salivary gland is extremely rare, it should be included in the differential diagnosis of poorly differentiated salivary gland neoplasms, especially with a fibromyxoid background. Pathologists should bear in mind that DSRCT may occur in major salivary glands and should perform immunohistochemistry with appropriate antibodies, not only those against keratin and desmin, but also one detecting the C-terminal region of WT-1. Furthermore, molecular detection of *EWSR1-WT1* fusion gene conclusively confirmed the diagnosis of DSRCT in this uncommon location.

## Background

Desmoplastic small round cell tumor (DSRCT) is rare and a highly aggressive neoplasm that typically involves the soft tissues of the abdomen or pelvis in children or young adults, showing a male predilection. Although it occurs over a wide age range, the peak incidence is in the third decade of life. DSRCT usually shows widespread abdominal serosal involvement, and overall patient survival is poor. On the other hand, extra-abdominal DSRCT is very rare. Previous cases have been reported to arise in the lung [1], pleura [2], paranasal sinuses [3], central nervous system [4], and scalp soft tissue [5] . DSRCT in major salivary glands has been reported, but it is extremely rare. To our knowledge, only 5 cases occurring in the salivary gland have been reported in the English literature [6–10]. Here, we report a case of a primary parotid gland DSRCT in a 49-year-old man, who is the oldest patient known to have been affected by this tumor, and who has survived for 3 years without recurrence. We also summarize the clinicopathological features of DSRCT in the salivary gland.

## Case presentation

### Clinical features

**Fig. 1:**
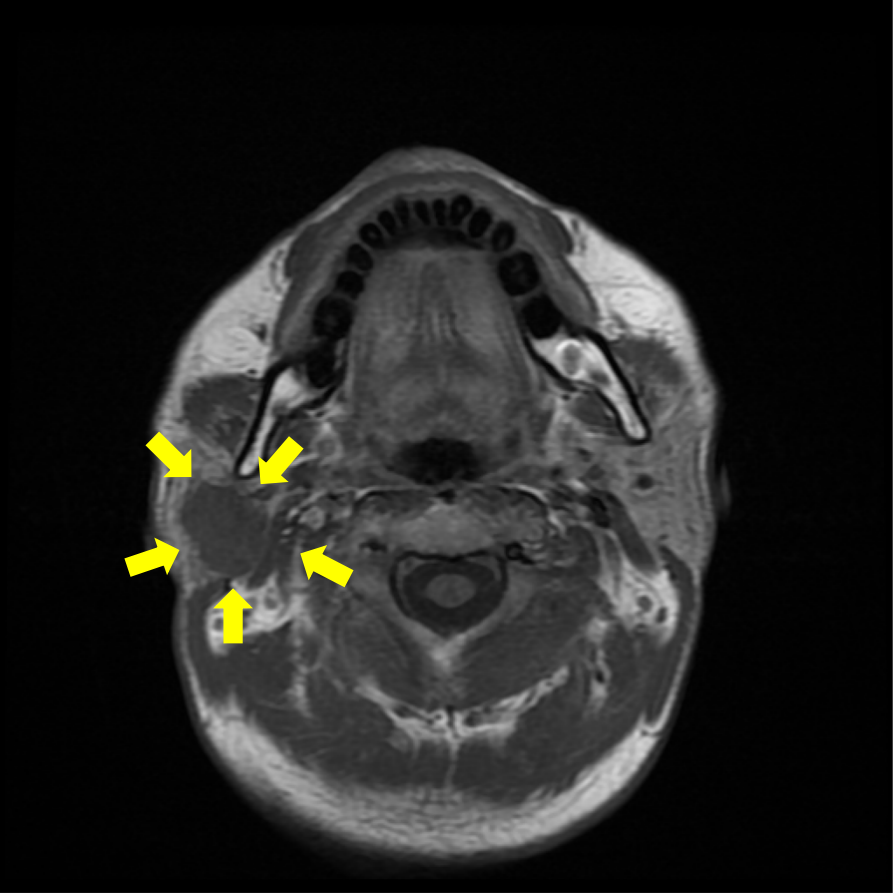
T1-weighted magnetic resonance imaging (MRI) showing a low-enhanced, well-defined mass in the right parotid gland (arrow)

A 49-year-old Japanese man noticed a mass in the right parotid gland without pain. There was no history of weight loss, fever, or night sweats. Ultrasound examination demonstrated that the tumor was a solid mass about 2 cm in diameter. T1-weighted magnetic resonance imaging showed a low-intensity, well-defined mass in the right parotid gland unaccompanied by lymph node swelling (Fig. 1). Abdominal computer tomography (CT) and whole-body positron emission tomography (PET) scan revealed no other tumor elsewhere. Although fine-needle aspiration was performed several times, it was difficult to obtain tumor cells for diagnosis, except for cells from normal salivary glands. Superficial parotidectomy was therefore performed and the tumor was successfully resected without facial nerve paralysis. After parotidectomy, the patient received radiotherapy and is currently alive and well with no evidence of recurrence after 3 years.

### Pathological findings

**Fig. 2:**
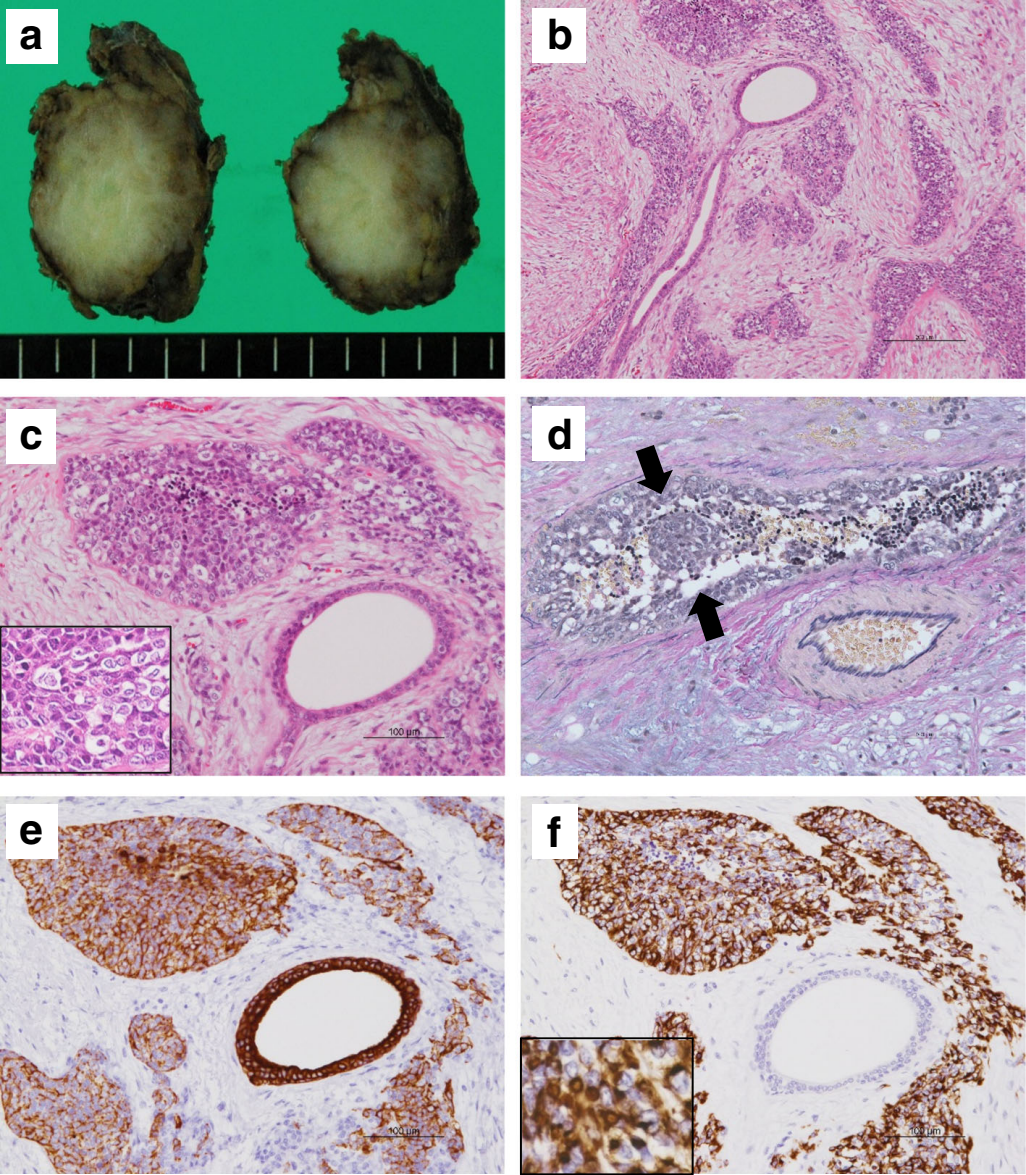
Macroscopic and histological findings of the salivary gland tumor. **a** The resected right parotid gland mass, appearing as a firm tan-colored tumor. **b** Well-defined nests of tumor cells varying in size are separated by a fibromyxoid stroma. (Hematoxylin-eosin, scale bar: 200 μm). **c** The nests are composed of small blue cells with scant cytoplasm and medium-sized cells with clear cytoplasm (insert). The normal salivary glands can be seen adjacent to the tumor nests. (scale bar: 100 μm). **d** Venous invasion (arrows) is evident (Elastica van Gieson staining). **e** Immunohistochemistry of the tumor cells shows positivity for CK8/18. **f** The tumor cells also show diffuse cytoplasmic positivity for desmin, although focal dot-like paranuclear positivity is also seen (inset)

Grossly, the tumor occupied the superficial lobe of the right parotid gland, and was solid and firm, measuring 2.7 × 2.7 × 2.3 cm. It was well circumscribed without a fibrous capsule, and the cut surface was grayish tan in color showing some lobulation at the tumor borders (Fig. 2a). Macroscopic necrosis or intratumoral hemorrhage was not evident. Histologically, the tumor predominantly showed a border that was well-defined from the surrounding tissue, although it was focally infiltrative in some areas. There was no fibrous capsule around the tumor. The tumor was composed of sharply demarcated cellular nests of various-sizes, growing in a paucicellular fibromyxoid or collagenous stroma (Fig. 2b). The tumor cells were round to polygonal and small to medium-sized, with scant cytoplasm and hyperchromatic irregular round nuclei with granular chromatin (Fig. 2c). Tumor cells with clear cytoplasm were also found in tumor nests, but rhabdoid cells were not identified. Apoptotic bodies were occasionally found, but necrotic foci were not evident. Venous invasion was detected (Fig. 2d). Some non-neoplastic salivary ducts were found between tumor nests, demonstrating that the tumor infiltration had extended within the parotid parenchyma. There was no evidence of regional lymph node metastasis.

### Immunohistochemical findings

**Fig. 3:**
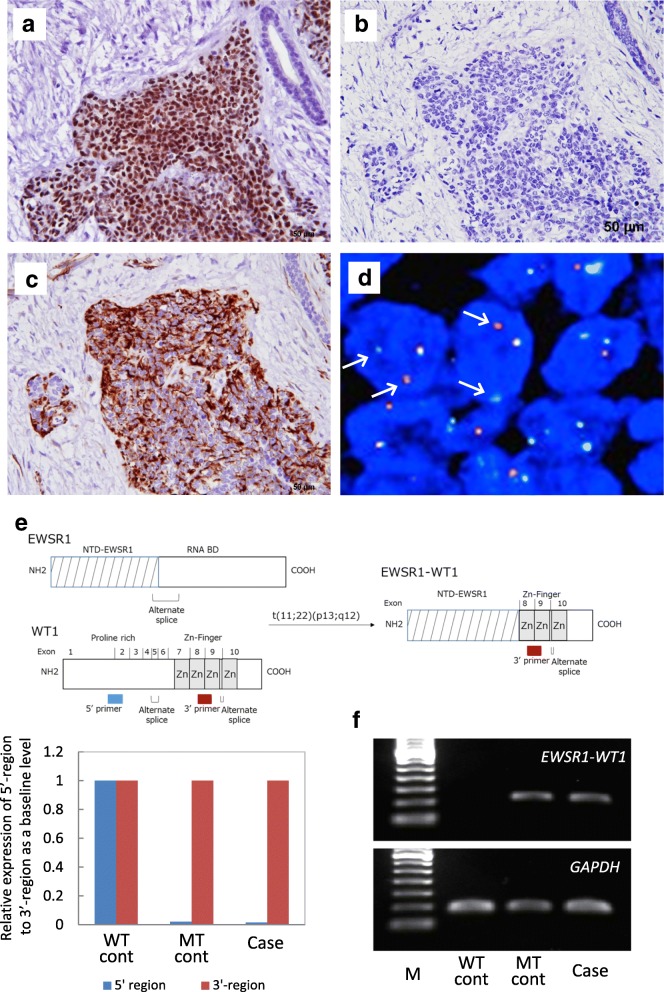
Immunohistochemical findings and molecular analyses of the salivary gland tumor. **a** C-WT1 shows nuclear positivity (scale bar: 50 μm). **b** N-WT1 (WT49) shows nuclear and cytoplasmic negativity. **c** N-WT1(6F-H2) shows cytoplasmic positivity. **d** FISH analysis using a break-apart probe for the EWSR1 gene region demonstrates the rearrangement in most of the cells. **e** The 3′/5′ expression imbalance assay based on RT-PCR reveals that the tumor lacked 5′-regional expression of the WT1 gene. The primers were designed to measure the expressions at two regions for each gene transcript: a 5′ probe pair located far upstream of the exons and a second pair located within the exons located further 3′ in the WT1 gene. PCR analysis was performed using these 5′ and 3′ primers, respectively. The Ct data were normalized to wild-type control tissue, and the normalized data was expressed as the relative gene expression level. WT cont; wild-type control (ovarian serous carcinoma), MT cont; mutant type control (typical DSRCT). **f** RT-PCR analysis showed that the EWS-WT1 fusion gene was present in the sample. M; marker, WT cont and MT cont; same as above

An immunohistochemical study was performed using formalin-fixed paraffin embedded (FFPE) sections of representative tumor blocks, using the antibodies summarized in Table 1. The tumor cells were positive for cytokeratin (AE1/AE3, CK8/18), EMA, vimentin, desmin, and focally positive for CD56 (Fig. 2e). Desmin immunoreactivity showed a diffuse cytoplasmic pattern for the most part, as well as a paranuclear dot-like pattern in a smaller proportion of the tumor (Fig. 2f). The cells were negative for chromogranin A, synaptophysin, S100 protein, CK5/6, p63, CD99 (MIC2), GFAP, and CD117 (KIT). They were also negative for calponin and α-smooth muscle actin (αSMA), in contrast to the positivity shown by myofibroblast-like spindle cells in the stroma. The Ki-67 labeling index was almost 50%. For WT1, the tumor cells showed strong nuclear staining with an antibody recognizing the C-terminal region of WT1 (C-WT1) (polyclonal, Abnova). However, neither of two N-terminal antibodies (N-WT1), WT49 (Leica) nor 6F-H2 (Dako), elicited positive nuclear staining, although the latter showed nonspecific cytoplasmic staining, (Fig. 3a-c).

**Table 1:** List of Antibodies

Antibody	Clone	Source	Dilution
AE1/AE3	E29	DAKO	1:500
CK8/18	5D3	Nobocastra	1:200
CK5/6	D5/16/B4	DAKO	1:400
EMA	E29	DAKO	1:500
vimentin	V9	Roche	RTU
desmin	DER11	Roche	RTU
αSMA	1A4	DAKO	1:300
calponin	hCP	SIGMA	1:30000
chromogranin A	LK2H10	Roche	RTU
synaptophysin	27G12	Leica	RTU
S100 protein	polyclonal	DAKO	1:5000
CD56	CD564	Leica	RTU
p63	7JUL	Leica	RTU
CD99 (MIC-2)	12E7	DAKO	1:100
GFAP	polyclonal	DAKO	1:2000
CD117 (c-Kit)	polyclonal	DAKO	1:500
Ki-67	MIB1	DAKO	1:200
WT1	polyclonal	Abnova	1:5000
WT1	WT49	Leica	1:100
WT1	6F-H2	DAKO	1:100

*αSMA* α-smooth muscle actin, *EMA* epithelial membrane antigen, *WT1* Wilms tumor 1, *GFAP* glial fibrillary acidic protein, *RTU* ready-to-use

#### Molecular analyses

Dual-colored fluorescence in situ hybridization (FISH) analysis using *EWSR1* break-apart probes (Abbott Molecular, Abbott Park, IL) on FFPE tissue detected *EWSR1* split signals in 94% of the tumor cells (Fig. 3d). We then performed 3′/5′ expression imbalance assay based on reverse transcription polymerase chain reaction (RT-PCR) according to the methods described by Suehara et al [11], in order to determine whether aberrant *WT1* gene expression was present. The result clearly showed that 5′-regional expression of the *WT1* gene was lacking in the tumor (Fig. 3e), being consistent with absence of immunoreactivity with the N-WT1 antibody at the protein level revealed by immunohistochemistry (Fig. 3b-c). To confirm these gene alterations, RT-PCR for the *EWSR1-WT1* fusion gene was performed using a forward primer (5′-TCCTACAGCCAAGCTCCAAGT-3′, *EWSR1* exon 7) and reverse primer (5′-ACCTTCGGTTCACAGTCCTTG-3′, *WT1* exon 8) [12]. This revealed the characteristic *EWSR1-WT1* fusion gene (Fig. 3f).

## Discussion

DSRCT is an uncommon malignant neoplasm that first described in two boys in 1989 [13]. DSRCT occurs mainly in the abdominal cavity, retroperitoneum, and pelvis, but Gerald et al. reported that 6% of DSRCTs can occur in an extra-abdominal location [14]. Histologically, DSRCT is characterized by various-sized nests composed of small neoplastic cells with a prominent desmoplastic, fibromyxoid, or collagenous stroma. Immunohistochemically, DSRCT shows a distinctive and characteristic pattern of multi-phenotypic differentiation. Tumor cells express proteins associated with epithelial, muscular and neural differentiation. The distinctive dot-like staining pattern of desmin is typical, but a diffuse cytoplasmic pattern has also been described [7], as seen in the present case. Hill et al. reported that nuclear immunoreactivity with C-WT1 antibody, which was observed in this case and is considered attributable to *EWSR1-WT1* gene fusion, is useful for differentiating DSRCT from other small blue cell tumors, such as Ewing sarcoma [9]. The variable immunohistochemical reactivity of WT1 protein according to the anti-WT1 antibody employed may be a diagnostic pitfall. Obviously, it is critically important to use C-WT1 antibody for diagnosis of DSRCT, because N-WT1 antibody would give negative results, as was confirmed by the lack of the 5′-regional expression of the *WT1* gene in the present case.

Although the present case showed typical histological features and immunohistochemical profiles, successful detection of the *EWSR1-WT1* gene rearrangement involving t(11,22)(q13;q12) by FISH and RT-PCR assays using FFPE tissues conclusively confirmed the diagnosis of DSRCT in this uncommon location.

The clinicopathological features of salivary gland DSRCT reported previously in the English literatures are summarized in Table 2. All 6 cases, including the present one, occurred in males, and the age at the diagnosis ranged from 5 to 49 years, with a median age of 28 years. Two of these cases occurred in the fifth decade, although DSRCT is generally considered as a differential diagnosis in children or young adults. The tumors ranged in size from 2.7 to 5 cm, with an average of 4.1 cm. Among the six patients, three were still undergoing follow-up, and one had died due to systemic metastasis. Salivary gland DSRCT showed histological features similar to those of abdominal DSRCT. The overall survival in abdominal DSRCT patients is generally poor, despite multimodality therapy [15], According to the present case as well as previous cases, only one among 6 patients with salivary gland DSRCT died due to metastasis. The prognosis of salivary gland DSRCT is unclear because of its rarity, but early detection or complete resection of salivary gland DSRCT might result in better prognosis.

**Table 2:** Clinicopathological summary of Major Salivary Gland DSRCT

Authors	Age(y) Sex	Salivary gland	Size (cm)	IHC+	Molecular test	Lymph node metastasis	Resection margin	Additional therapy after operation	Outcome (mo)
Pang et al	41/M	Left submandibular	5	Desmin, EMA, CK, WT1, CD56	FISH, RT-PCR	Positive	Negative	ND	1 (DOC)
Yin et al	24/M	Right submandibular	4	Desmin, Vimentin, CK, NSE, p53	FISH, RT-PCR	NS	Negative	Chemo, RT	7 (AFD)
Cho et al	26/M	Left sumandibular	4	Desmin, CK, NSE, Vimentin	RT-PCR	Positive	NS	Chemo, RT	23 (DOD) systemic metastasis
Hill at al	5/M	Parotid	NS	Desmin, EMA, WT1, CK, NSE, vimentin	FISH, RT-PCR, Southern blot	NS	NS		NS
Wolf et al	23/M	Left parotid	5	Desmin, EMA, CK, NSE	FISH	Negative	Positive	Chemo, RT	10 (AFD)
Present case	49/M	Right parotid	2.7	Desmin, EMA, CK, WT1, vimentin, CD56	FISH、RT-PCR	Negative	Positive	RT	36 (AFD)

*IHC* immunohistochemistry, *mo* month, *EMA* epithelial membrane antigen, *CK* cytokeratin, *WT1* Wilms tumor 1, *NSE* neuro-specific enolase, *PR* progesterone receptor, *NS* not specified, *ND* not done, *Chemo* chemotherapy, *RT* radiotherapy, *DOC* died of other complication, *AFD* alive and free of tumor, *DOD* died of disease

The origin of DSRCT remains unclear. It has been speculated that DSRCTs are derived from mesothelial or submesothelial cells because a vast majority of patients develop DSRCTs in cavities that are lined with mesothelial cells or because tumor cells show immunohistochemical positivity for epithelial and mesenchymal antigens including desmin, and WT-1 [12].

DSRCT is not usually included in the differential diagnosis in primary salivary gland tumors in view of its rarity. Because DSRCT is composed of small nests with cohesive small to medium-sized cells and shows immunoreactivity for epithelial markers, it might be diagnosed as carcinomas, such as small cell carcinoma, poorly differentiated carcinoma, undifferentiated carcinoma, without staining for desmin. Primary small cell carcinoma of the salivary glands is also rare, accounting for approximately 2% of all salivary gland tumors, and most arise in patients 50 years old or more [16]. Positivity for synaptophysin or chromogranin is useful for identification of small cell carcinoma. It is also difficult to rule out poorly differentiated carcinoma or undifferentiated carcinoma, although an abundant desmoplastic or fibromyxoid stroma is an unusual feature in these carcinomas. Therefore, this might be a diagnostic clue for DSRCT. Other differential diagnosis, such as malignant melanoma, metastatic neuroblastoma, or lymphoma, are excluded with positivity for epithelial markers. Merkel cell carcinoma is also the differential diagnosis, but it is excluded with staining for neuroendocrine markers and its characteristic dot-like staining pattern for cytokeratin 20.

Primary non-lymphoid mesenchymal neoplasms of major salivary glands are also rare and account for only 1.9–5% of cases [8]. Most mesenchymal tumors are benign. The most common mesenchymal tumor is lipoma, [8] [17]. Malignant cases are extremely rare [8]. Because of the histological features of small round cell tumors, Ewing sarcoma and rhabdomyosarcoma are also included as differential diagnoses. Positivity for desmin and WT1 nuclear staining in addition to positive epithelial markers strongly favors a diagnosis of DSRCT over that of Ewing sarcoma and rhabdomyosarcoma. Furthermore, molecular biology studies are useful and important in differentiating DSRCT from Ewing sarcoma and rhabdomyosarcoma. In the present case, the tumor nests were composed of not only small-sized cells with a high nuclear/cytoplasmic ratio, but also medium-sized cells with clear cytoplasm. These cells with clear cytoplasm were also described in a previous report [6]. Because both two cell types share the same immunohistochemical profiles in the present case, cells with clear cytoplasm should also be recognized as neoplastic, and not as non-neoplastic bystander cells such as myoepithelial cells.

## Conclusions

In summary, we have reported a rare primary DSRCT with venous invasion arising from the parotid glands of a middle-aged man. It is very important to be aware of the fact that DSRCT may occur in major salivary glands. To ensure accurate diagnosis with immunohistochemistry, desmin and the C-terminal region of WT1 are very useful markers in addition to epithelial markers. Furthermore, molecular detection of *EWSR1-WT1* fusion gene conclusively confirmed the diagnosis of DSRCT in this uncommon location.

## Abbreviations

CK: cytokeratin
CT: Computed tomography
C-WT1: WT1 antibody detecting the C-terminal region
DSRCT: Desmoplastic small round cell tumor
FFPE: formalin-fixed paraffin embedded
FISH: fluorescence in situ hybridization
GFAP: glial fibrillary acid protein
N-WT1: WT1 antibody detecting the C-terminal region
PET: positron emission tomography
RT-PCR: reverse transcription polymerase chain reaction
RTU: ready-to-use
SMA: smooth muscle actin
WT1: Wilms tumor 1
